# Deep learning assessment of breast terminal duct lobular unit involution: Towards automated prediction of breast cancer risk

**DOI:** 10.1371/journal.pone.0231653

**Published:** 2020-04-15

**Authors:** Suzanne C. Wetstein, Allison M. Onken, Christina Luffman, Gabrielle M. Baker, Michael E. Pyle, Kevin H. Kensler, Ying Liu, Bart Bakker, Ruud Vlutters, Marinus B. van Leeuwen, Laura C. Collins, Stuart J. Schnitt, Josien P. W. Pluim, Rulla M. Tamimi, Yujing J. Heng, Mitko Veta

**Affiliations:** 1 Medical Image Analysis Group, Department of Biomedical Engineering, Eindhoven University of Technology, Eindhoven, The Netherlands; 2 Department of Pathology, Harvard Medical School, Beth Israel Deaconess Medical Center, Boston, Massachusetts, United States of America; 3 Division of Population Sciences, Dana Farber Cancer Institute, Boston, Massachusetts, United States of America; 4 Division of Public Health Sciences, Department of Surgery, Washington University School of Medicine and Alvin J. Siteman Cancer Center, St Louis, Missouri, United States of America; 5 Philips Research Europe, High Tech Campus, Eindhoven, The Netherlands; 6 Dana-Farber/Brigham and Women’s Cancer Center, Harvard Medical School, Dana-Farber Cancer Institute-Brigham and Women’s Hospital, Boston, Massachusetts, United States of America; 7 Channing Division of Network Medicine, Department of Medicine, Harvard Medical School, Brigham and Women’s Hospital, Boston, Massachusetts, United States of America; University of Central Florida (UCF), UNITED STATES

## Abstract

Terminal duct lobular unit (TDLU) involution is the regression of milk-producing structures in the breast. Women with less TDLU involution are more likely to develop breast cancer. A major bottleneck in studying TDLU involution in large cohort studies is the need for labor-intensive manual assessment of TDLUs. We developed a computational pathology solution to automatically capture TDLU involution measures. Whole slide images (WSIs) of benign breast biopsies were obtained from the Nurses’ Health Study. A set of 92 WSIs was annotated for acini, TDLUs and adipose tissue to train deep convolutional neural network (CNN) models for detection of acini, and segmentation of TDLUs and adipose tissue. These networks were integrated into a single computational method to capture TDLU involution measures including number of TDLUs per tissue area, median TDLU span and median number of acini per TDLU. We validated our method on 40 additional WSIs by comparing with manually acquired measures. Our CNN models detected acini with an F1 score of 0.73±0.07, and segmented TDLUs and adipose tissue with Dice scores of 0.84±0.13 and 0.87±0.04, respectively. The inter-observer ICC scores for manual assessments on 40 WSIs of number of TDLUs per tissue area, median TDLU span, and median acini count per TDLU were 0.71, 0.81 and 0.73, respectively. Intra-observer reliability was evaluated on 10/40 WSIs with ICC scores of >0.8. Inter-observer ICC scores between automated results and the mean of the two observers were: 0.80 for number of TDLUs per tissue area, 0.57 for median TDLU span, and 0.80 for median acini count per TDLU. TDLU involution measures evaluated by manual and automated assessment were inversely associated with age and menopausal status. We developed a computational pathology method to measure TDLU involution. This technology eliminates the labor-intensiveness and subjectivity of manual TDLU assessment, and can be applied to future breast cancer risk studies.

## Background

Most benign breast lesions and breast cancers arise in the terminal duct lobular units (TDLUs) [1], the milk-producing structures of the breast. Russo *et al*. [2] historically classified TDLUs into four lobule types: type 1 (least developed; <12 acini/lobule), type 2 (evolves from type 1; intermediate in degree of differentiation; between 12 and 80 acini/lobule), type 3 (fully developed structures; >80 acini/lobule), and type 4 (occurs during pregnancy and lactation). Pathologists have used these qualitative lobule types to evaluate TDLU involution indicated by the presence of more type 1 lobules and less type 2 and 3 lobules after the completion of childbearing and with physiological aging [3]. In quantitative terms, TDLU involution is characterized by a reduction of the size of TDLUs, the number of acini, and the number of acini per TDLU [4–8]. Previous work by our group and others evaluated TDLU involution using qualitative measures and reported that women with less TDLU involution (i.e., majority of lobules were of types 2 and 3) were more likely to develop breast cancer compared to those with predominantly type 1 lobules independent of age [5, 9, 10, 11]. Thus, TDLU involution measures may be utilized as a biomarker to assess breast cancer risk [9, 10].

Efforts to develop quantitative measures of TDLU involution started with McKian *et al*. [11] who evaluated the number of acini and TDLU area on histopathological sections. Rosebrock *et al*. [12] were the first to automatically estimate quantitative measurements from TDLUs and use those measurements to describe and classify them. Later, Figueroa *et al*. standardized three quantitative measures of TDLU involution—number of TDLUs per tissue area (TDLUs/mm^2^), median TDLU span, and the median number of acini per TDLU (median acini/TDLU)—by assessing up to 10 TDLUs in the normal tissue for a WSI [4, 10, 13, 14]. The examined tissue area was corrected for the amount of adipose tissue present. These quantitative measurements still relied on manual histological assessment of breast tissue, and remained subjective and labor-intensive. Thus, the need for manual qualitative and/or quantitative assessment by pathologists is a major bottleneck to studying TDLU involution in large epidemiological studies.

Automated image analysis methods have the potential to decrease the workload of pathologists and standardize clinical practice [15]. Known or novel tissue biomarkers can now be automatically quantified [15–20] and deep learning has also been applied to recognize morphological tissue patterns for diagnostic purposes [21–27]. More specifically, networks have been successfully developed for tasks in breast histopathology [28–33]. Most recently, state-of-the-art deep convolutional neural networks (CNN) have been shown to outperform pathologists in detecting metastases in sentinel lymph nodes of breast cancer patients [34]. In this study, we developed an automated method to quantitatively assess TDLU involution. First, we constructed and optimized three deep neural networks to detect and/or segment acini, TDLUs, and adipose tissue. These three networks were integrated into a single method to compute TDLU involution measures. Our automated method was validated by comparing the automated measures with manually acquired measures on an independent set of images.

## Methods

### Subjects and acquisition of images

The participants in this study are from the Nurses’ Health Study (NHS) and NHSII. The NHS was established in 1976 with 121,700 US female registered nurses between 30–55 years of age, and NHSII was established in 1989 (n = 116,429, ages 25–42). All NHS/NHSII participants are followed up biennially to obtain updated information on a range of epidemiological data and identify newly diagnosed diseases [35]. Hematoxylin and eosin (H&E) breast tissue slides were retrieved for women who reported a biopsy-confirmed benign breast disease (BBD) and gave permission to review their biopsy records and original H&E slides [36–42]. The tissue was prepared and stained at the local centers and centrally reviewed. BBD H&E whole slide images (WSIs) were obtained by scanning the slides at ×40 magnification with a resolution of 0.16 *μm* per pixel using Pannoramic SCAN 150 (3DHISTECH Ltd, Budapest, Hungary). The study protocol was approved by the institutional review boards of the Brigham and Women’s Hospital and Harvard T.H. Chan School of Public Health, and those of participating registries as required. Informed consent was obtained from all NHS/NHSII participants.

### Developing the automated method for TDLU involution measures

In total, 92 WSIs from 92 benign breast biopsies from 67 pre- and 25 post-menopausal women were randomly selected from the NHS database. To capture the large variability in lobule sizes, pre-menopausal women were over selected to obtain data/annotation/ground truth for type 2 and 3 lobules since post-menopausal tissues were predominantly type 1 lobules. Due to the more challenging nature of the TDLU segmentation task, 92 WSIs were used to develop the TDLU segmentation neural network model while a subset of 50 out of the 92 WSIs was adequate to develop the acini detection and adipose tissue segmentation neural network models. Breast tissue with more adipose tissue has fewer TDLUs and acini [4], which influences the outcomes of TDLU involution measures (e.g. number of TDLUs per tissue area). Therefore, the adipose tissue model was developed to estimate and account for the percentage of adipose tissue.

**Fig 1:**
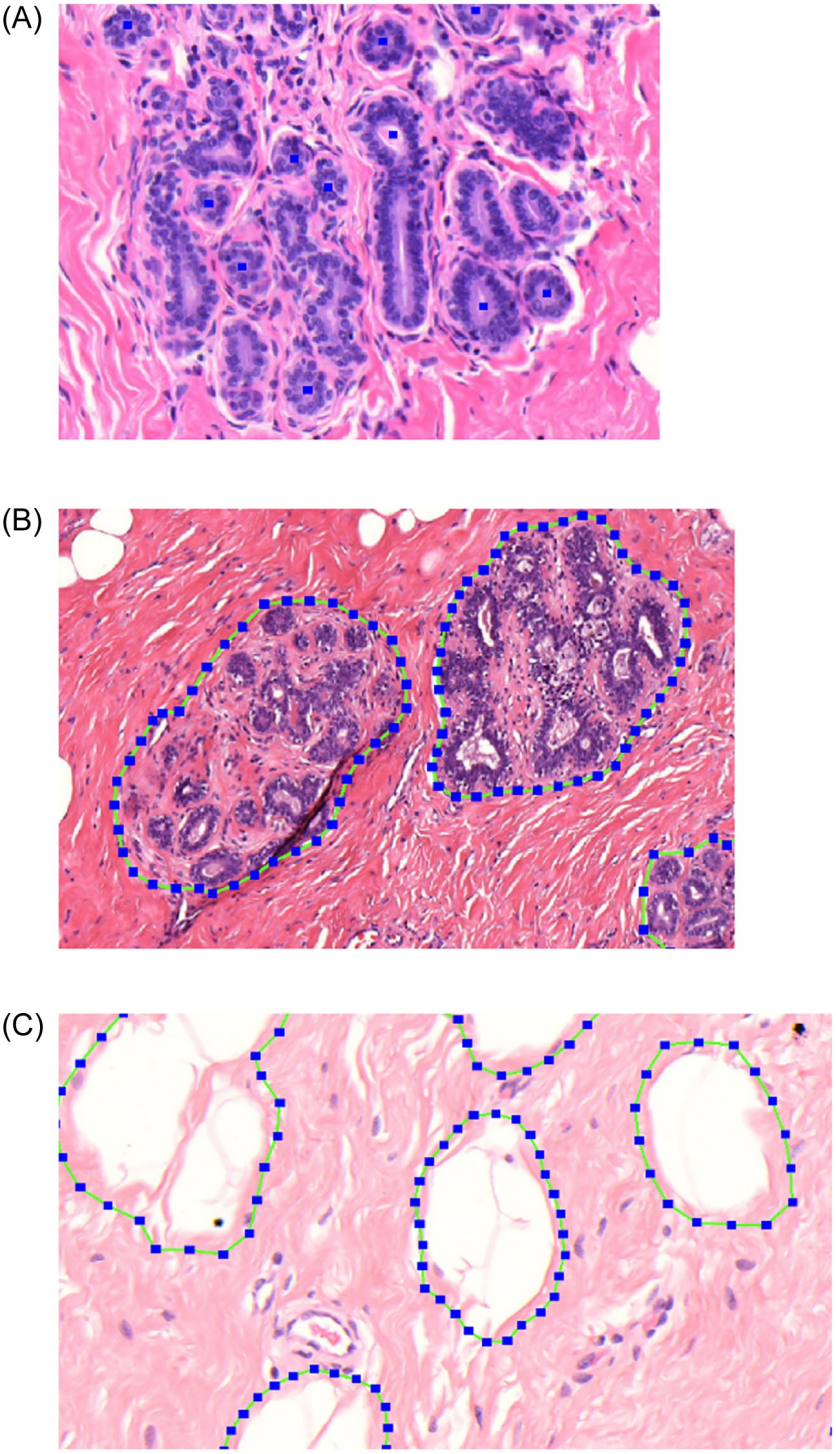
Examples of annotations for acini (A; annotated by blue squares), terminal duct lobular units (B) and adipose tissue (C).

TDLUs, acini, and adipose tissue were annotated within a region of interest (ROI) comprising approximately 10%, 10%, and 2.5% of the total tissue area, respectively. Annotation was done using the open-source software Automated Slide Analysis Platform (ASAP; Computation Pathology Group, Radboud University Medical Center). TDLUs were defined as clusters of acini in a lobular configuration. TDLU boundary was defined by the non-specialized/extra-lobular stroma. In order to assess involution in histologically normal breast parenchyma only, TDLUs with proliferative or metaplastic changes were not annotated as TDLUs but remained as background. Acini were defined as small spherical structures lined by epithelial cells and surrounded by myoepithelial cells. Acini with elongated shapes, epithelial proliferation, apocrine metaplasia, or without lumina were not annotated. In total, 25,645 acini and 1,631 TDLUs were annotated. Fig 1 shows examples of annotated acini, TDLUs and adipose tissue.

Acini, TDLUs, and adipose tissue were detected and segmented using the U-Net CNN architecture [43, 44]. Since we had different datasets for the three tasks, three separate models were trained. To construct the acini and adipose networks and evaluate the performance, the 50 annotated WSIs were split into 5 sets of 10 WSIs for cross validation. In each of the 5 folds, training was done on 30 WSIs (60%), validation on 10 WSIs (20%), and testing on 10 WSIs (20%). Annotated WSIs to construct the TDLU network were split into 9 sets of 10 or 11 WSIs for cross validation. For each of the 9 folds, training was done on 7 sets (~78%), validation on 1 set (~ 11%), and testing on 1 set (~ 11%). For all three methods, the model results from the first dataset split was used in all subsequent experiments (the models from the remaining folds are only used to evaluate the performance of the individual methods). All CNN models are described in the S1 Methods, and the acini detection network has been previously described [29]. To assess whether the training sets were large enough to learn to detect acini and segment TDLU ablation experiments were performed.

The three individual networks were integrated into a single automated method. This method can determine the three standardized quantitative measures by Figueroa *et al*. (i.e., TDLUs/mm^2^, median TDLU span (μm), and median acini/TDLU [4, 10, 13, 14]) as well as two additional quantitative measures: number of acini per tissue area (acini/mm^2^) and median TDLU area (mm^2^). Our method can also perform TDLU involution assessment using qualitative categories as described by Russo *et al*. [2] (i.e., predominant lobule type 1, 2 or 3) and Baer *et al*. [9] (i.e., no type 1 lobules, predominantly type 1 and no type 3, and mixed lobules (all others)). Thus, in total, our automated method can capture five quantitative and two qualitative measures of TDLU involution.

### Validating the automated measures of TDLU involution

We validated our automated method by comparing automated results with manual assessment on an independent set of 40 WSIs (Table 1). Sixty WSIs were initially chosen at random from the NHS/NHSII BBD cases to contain 30 pre- and 30 post-menopausal women. Upon further review, we excluded one woman who had type 4 lobules which suggests that she was pregnant or lactating at time of BBD diagnosis. By excluding type 4 lobules, our method is generalizable to non-pregnant/not lactating women.

**Table 1:** Demographic table of 40 participants used to validate the automated measures of TDLU involution.

	Pre-Menopausal	Post-Menopausal
*n*	20	20
Cohort, *n (%)*		
Nurses’ Health Study	5 (25)	12 (60)
Nurses’ Health Study II	15 (75)	8 (40)
Year of benign breast disease diagnosis, *n (%)*		
≥1978 to <1988	3 (15)	4 (20)
≥1988 to <1998	16 (80)	12 (60)
≥1998 to 2000	1 (5)	4 (20)
Age at benign breast disease diagnosis, *n (%)*		
30 to 39	8 (40)	1 (5)
40 to 49	10 (50)	6 (30)
50 to 59	2 (10)	6 (30)
≥60	0 (0)	7 (35)

For manual assessment (n = 59 WSIs), two observers assessed the three standardized quantitative measures. Each observer randomly selected a ROI of approximately 50 mm^2^ that contained an adequate number of normal TDLUs [4]. Within the ROI, the observers estimated the percentage of breast tissue (0 to 100%) and tissue containing adipose cells (<25%, 25–50%, 50–75%, or >75%), counted the total number of TDLUs, and randomly selected up to 10 normal TDLUs to measure span (μm) and count the number of spherical acini. TDLU boundary was defined by non-specialized/extra-lobular stroma. TDLUs were not counted if >50% of their acini were dilated by 2- to 3- fold, had metaplastic changes, or displayed ductal hyperplasia. TDLUs with <50% dilated acini were included and the acini within these TDLUs were counted (including dilated ones). Acini with elongated shape or no lumen were excluded. Three observers performed qualitative assessments using predominant lobule type by Russo *et al*. [2] and categories by Baer *et al*. [9]. For intra-observer evaluation, 10 out of 40 WSIs were randomly chosen for re-assessment.

Preliminary analyses of the 59 WSIs showed that although the manual and automated TDLU assessments were highly correlated, the values of the automated results for the number of acini per TDLU were lower than manual results. Therefore, we randomly selected 19 WSIs and linear regression to derive calibration weights based on the manual results to adjust our automated results. This calibration produced more meaningful values for interpretation. We applied the calibration weights to our automated results on the remaining 40 WSIs. The calibration coefficient to adjust the automated number of acini per TDLU measure to the manual results was found to be 3.888. The intercept was not significantly different from zero. We applied the calibration coefficient to our automated results on the remaining 40 WSIs by multiplying all median number of acini per TDLU outcomes by 3.888.

Tissue area was adjusted for the percentage of adipose tissue by multiplying the total tissue area by the percentage of non-adipose tissue. Since manual observers only estimated adipose tissue percentage in categories (<25%, 25–50%, 50–75%, or >75%), we used the center bin values for this multiplication.

### Association of TDLU measures with age and menopausal status

We also assessed manual and automated TDLU involution measures with age and menopausal status in the final 40 cases. This was to confirm that our measures were reflective of TDLU involution, as older women were expected to have more involution.

### Statistical analysis

The evaluation of the acini detection neural network model was done using the F1 score and the evaluation of the TDLU and adipose tissue segmentation network models was done using the Dice similarity coefficient. F1 score is the harmonic mean of precision (i.e., sensitivity) and recall (i.e., positive predictive value), which assesses how accurate the automated detection compares with ground truth (i.e., manual annotation). The calculation for the Dice similarity coefficient is identical to F1 score, except it assesses the accuracy of the automated segmentation when compared to ground truth. The F1 score and Dice similarity coefficient are similar. Traditionally, when used to evaluate the detection performance this score is referred to as the F1 score and when used to evaluate the performance of a segmentation algorithm it is referred to as the Dice similarity coefficient.

Inter- and intra-observer agreements for quantitative measures were summarized using intraclass correlation coefficient (ICC). Two-way mixed effects, consistency, single rater (ICC (3,1)) was used. ICC values of <0.5, between 0.5 and 0.75, between 0.75 and 0.9, and >0.9 are indicative of poor, moderate, good, and excellent reliability, respectively [45]. Intra- and inter-observer agreements for qualitative measures were determined by Fleiss’ Kappa. For comparison with automated results, the consensus of the three observers was used. The consensus was determined by majority voting.

To determine the strength and direction of association of quantitative TDLU involution measures with age, Spearman’s rank correlation coefficient was used. The Kruskal-Wallis test was used to examine the differences between groups of qualitative measures and age. Mann-Whitney U and Chi-squared tests were used to assess the independence of quantitative and qualitative TDLU involution assessment with menopausal status. The scores for F1, Dice, and Fleiss’ Kappa range from 0 to 1, with 1 indicating perfect correlation. Analyses were performed using R and *p <* 0.05 was considered statistically significant. The ICC confidence intervals were calculated using the ICC function in the irr R package.

## Results

### Performances of individual networks and establishing the automated method

The F1 score of the acini detection method was 0.73±0.07 [29]. The TDLU and adipose tissue segmentation methods obtained Dice similarity coefficients of 0.84±0.13 and 0.87±0.04, respectively. Ablation experiments showed that the methods converged with increasing number of training samples (S1 Fig).

**Fig 2:**
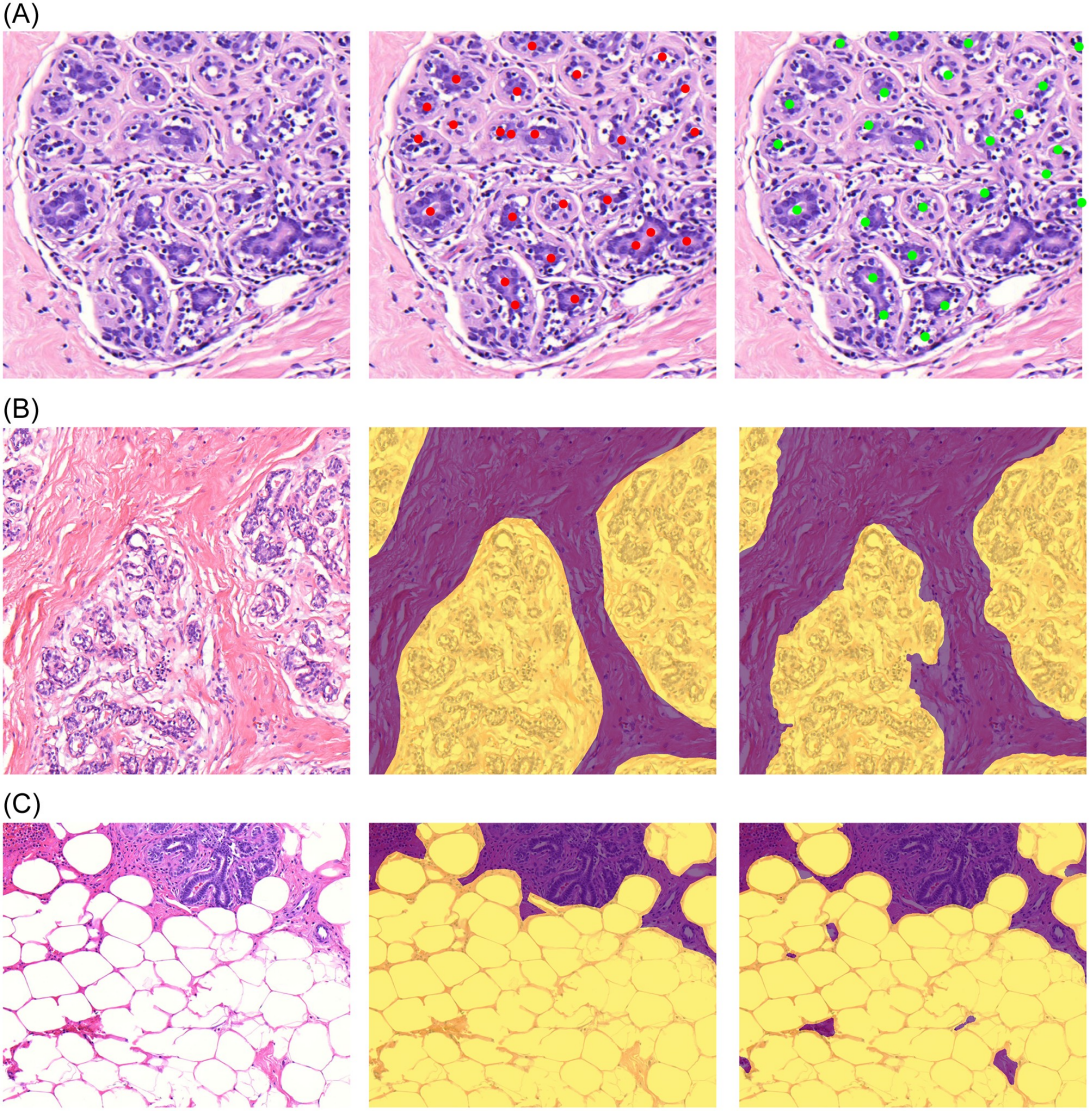
Results of the acini detection (A), terminal duct lobular unit (B), and adipose tissue (C) segmentation algorithms. The original images are in the left column, the middle column shows ground truth as annotated by human observers, and the detections and segmentations performed by the automated method are displayed in the right column.

**Fig 3:**
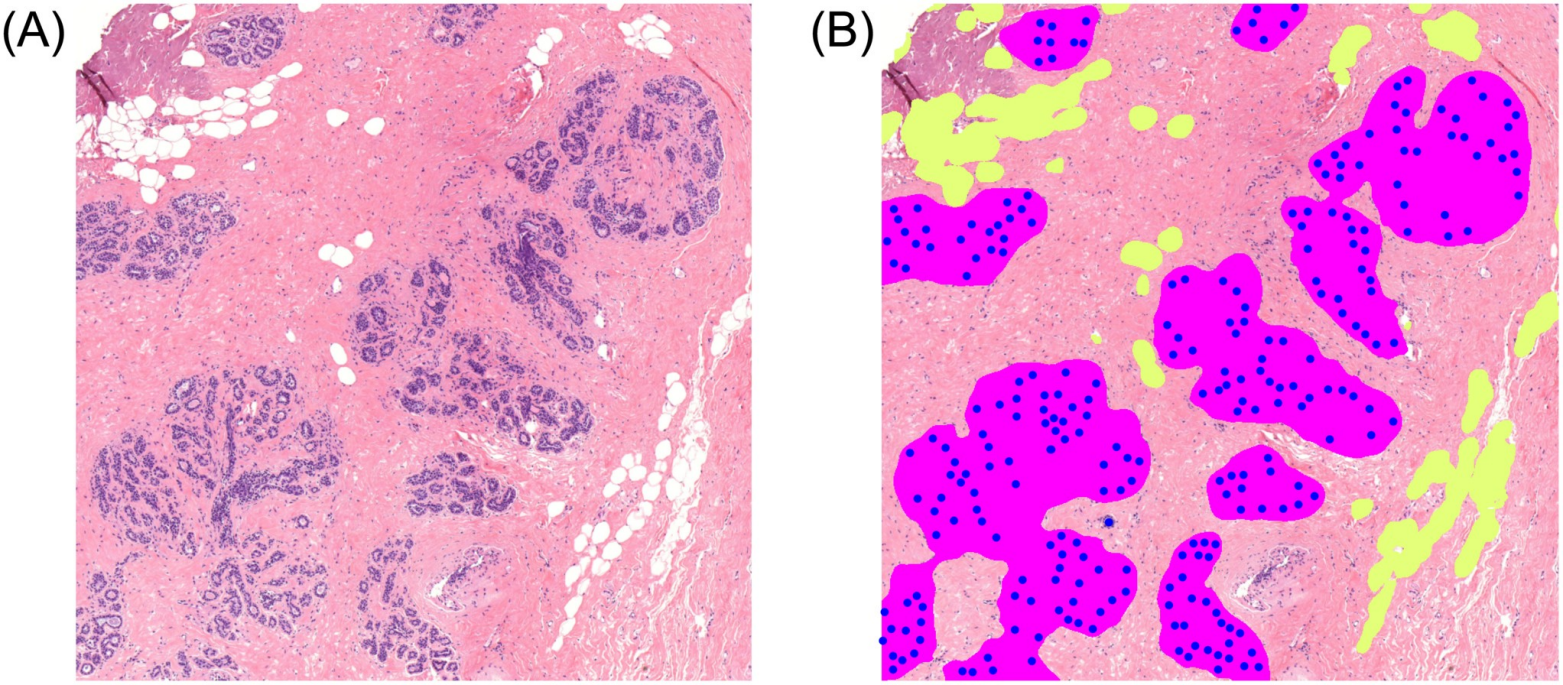
Results of the acini detection, terminal duct lobular unit, and adipose tissue segmentation algorithms (B) overlaid on the original image (A).

Based on this quantitative evaluation, which indicates good agreement, and subsequent qualitative assessment we determined that the performances of these three networks were adequate to be integrated into one automated method (Figs 2 and 3; S2 Fig).

The primary cause of discordance between manual assessment and the automated method was the detection of acini and TDLU with proliferative or metaplastic changes which were intentionally excluded from manual annotation. For example, in S2C Fig, our method incorrectly segments intraductal papillomas as TDLUs despite correctly identifying other TDLUs.

### Quantitative measures: Intra- and inter-observer agreement

Overall, quantitative measures derived from automated and manual methods achieved moderate to good inter-observer agreement (Table 2). The intra-observer agreement was good to excellent (ICC scores >0.8, 95% CI [0.53, 0.99]) and the inter-observer agreement among the two observers was moderate to good (ICC scores >0.7, 95% CI [0.51, 0.90]). The inter-observer agreement between the observers and the automated method was also moderate to good (ICC scores >0.5, 95% CI [0.19, 0.90]).

**Table 2:** Inter- and intra-observer intraclass correlation coefficient (ICC) scores and the 95% confidence interval (CI) for the quantitative terminal ductal lobular unit involution measures obtained from two observers and the automated method.

	Intra-observer ICC (95% CI)*		Inter-observer ICC (95% CI)^#^	
	Observer 1	Observer 2	Observer 1 vs 2	mean(observers) vs automated
Number of TDLUs per tissue area (mm^2^)	0.96 (0.86, 0.99)	0.82 (0.78, 0.98)	0.71 (0.51, 0.83)	0.80 (0.63, 0.90)
Median TDLU span (*μ*m)	0.91 (0.69, 0.98)	0.90 (0.67, 0.98)	0.81 (0.67, 0.90)	0.57 (0.19, 0.77)
Median number of acini per TDLU	0.91 (0.69, 0.98)	0.86 (0.53, 0.96)	0.73 (0.54, 0.85)	0.80 (0.62, 0.89)

*Intra-observer ICC was evaluated using 10 out of the 40 cases.

^#^Inter-observer ICC was evaluated using 40 cases.

### Qualitative measures: Intra- and inter-observer agreement

Qualitative measures between the three observers and the automated method achieved fair to moderate agreement (Table 3). Among the three observers, the inter-observer Kappa scores were fair to moderate (κ > 0.35 (*p*<0.01)) while there was a large variation in their intra-observer Kappa scores (κ from 0.048 (*p* = 0.880) to 1.000 (*p*<0.01)). The inter-observer agreement between the observers and the automated method was moderate (κ > 0.5 (*p*<0.01)). There was slightly more agreement in the evaluation of Russo *et al*. [2] predominant lobule type compared to Baer *et al*. [9] categories.

**Table 3:** Inter- and intra-observer Fleiss’ Kappa for qualitative terminal ductal lobular unit assessment among three observers using 40 and 10 cases, respectively.

	Intra-observer*						Inter-observer^#^			
	Observer 1		Observer 2		Observer 3		Observer 1,2 & 3		Consensus vote of observers vs automated	
	*κ*	p-value	*κ*	p-value	*κ*	p-value	*κ*	p-value	*κ*	p-value
Predominant lobular type										
by Russo *et al*. [2]	0.167	0.598	0.608	0.055	0.798	**0.012**	0.529	**<0.01**	0.536	**<0.01**
Lobular classification according to Baer *et al*. [9]	0.048	0.880	1.000	**<0.01**	0.798	**0.012**	0.370	**<0.01**	0.538	**<0.01**

*Intra-observer evaluation was done using 10 out of the 40 cases.

^#^Inter-observer evaluation was done using 40 cases.

### TDLU involution with age and menopausal status

**Fig 4:**
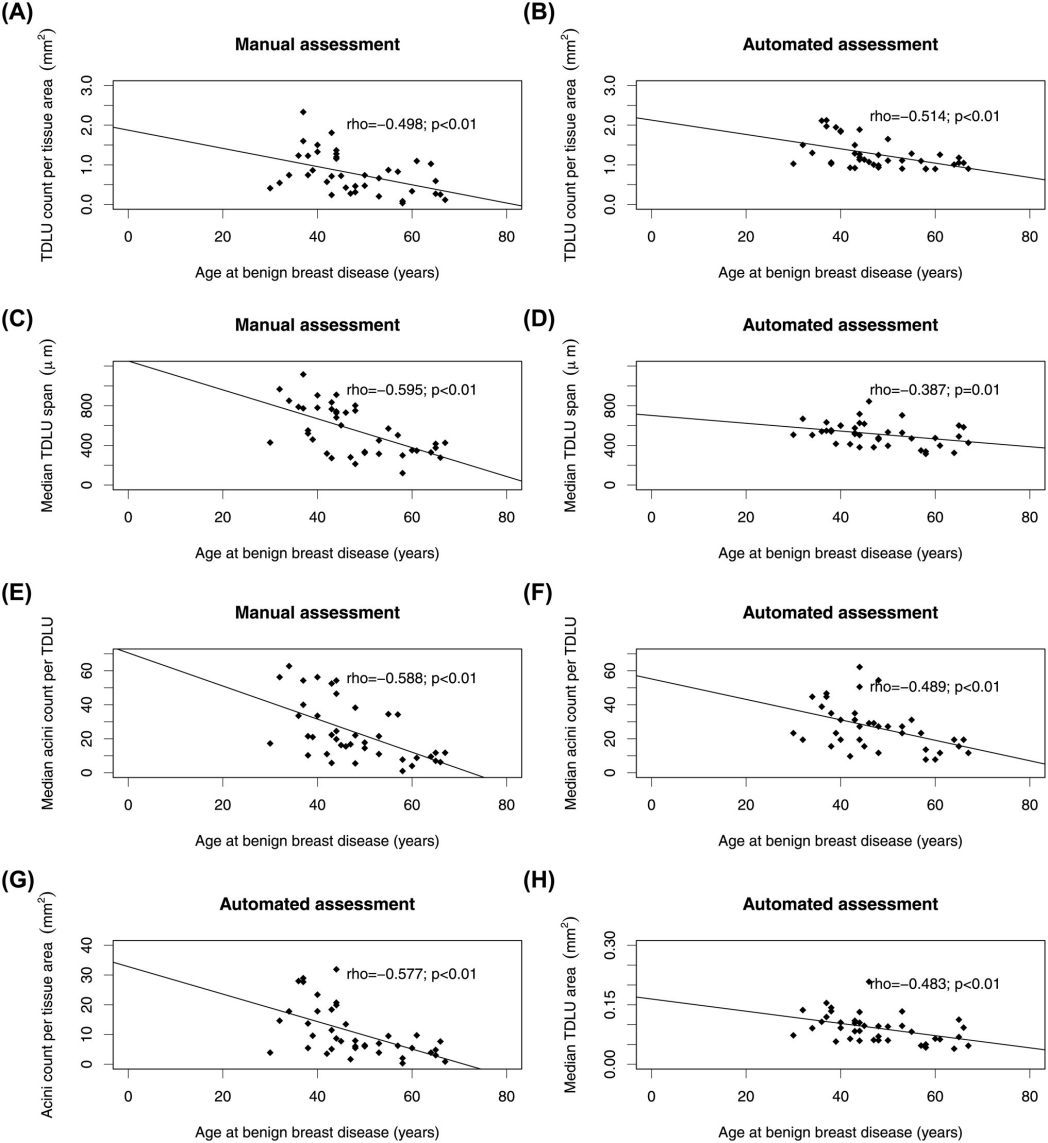
Scatterplots of the association of quantitative terminal ductal lobular unit (TDLU) involution measures and age. TDLU count per tissue area assessed using manual (**A**) and automated (**B**) method were significantly inversely correlated with age (*p*<0.01). Median TDLU span assessed manually (**C**) and with the automated method (**D**) was significantly inversely correlated with age (*p*<0.01 and *p* = 0.01). Median acini count per TDLU assessed using manual (**E**) and automated (**F**) assessment was also significantly inversely correlated with age (*p*<0.01). Acini count per tissue area assessed by the automated method was significantly inversely correlated with age (**G**; *p*<0.01). Median TDLU area assessed by the automated method was significantly inversely correlated with age (**H**; *p*<0.01).

**Fig 5:**
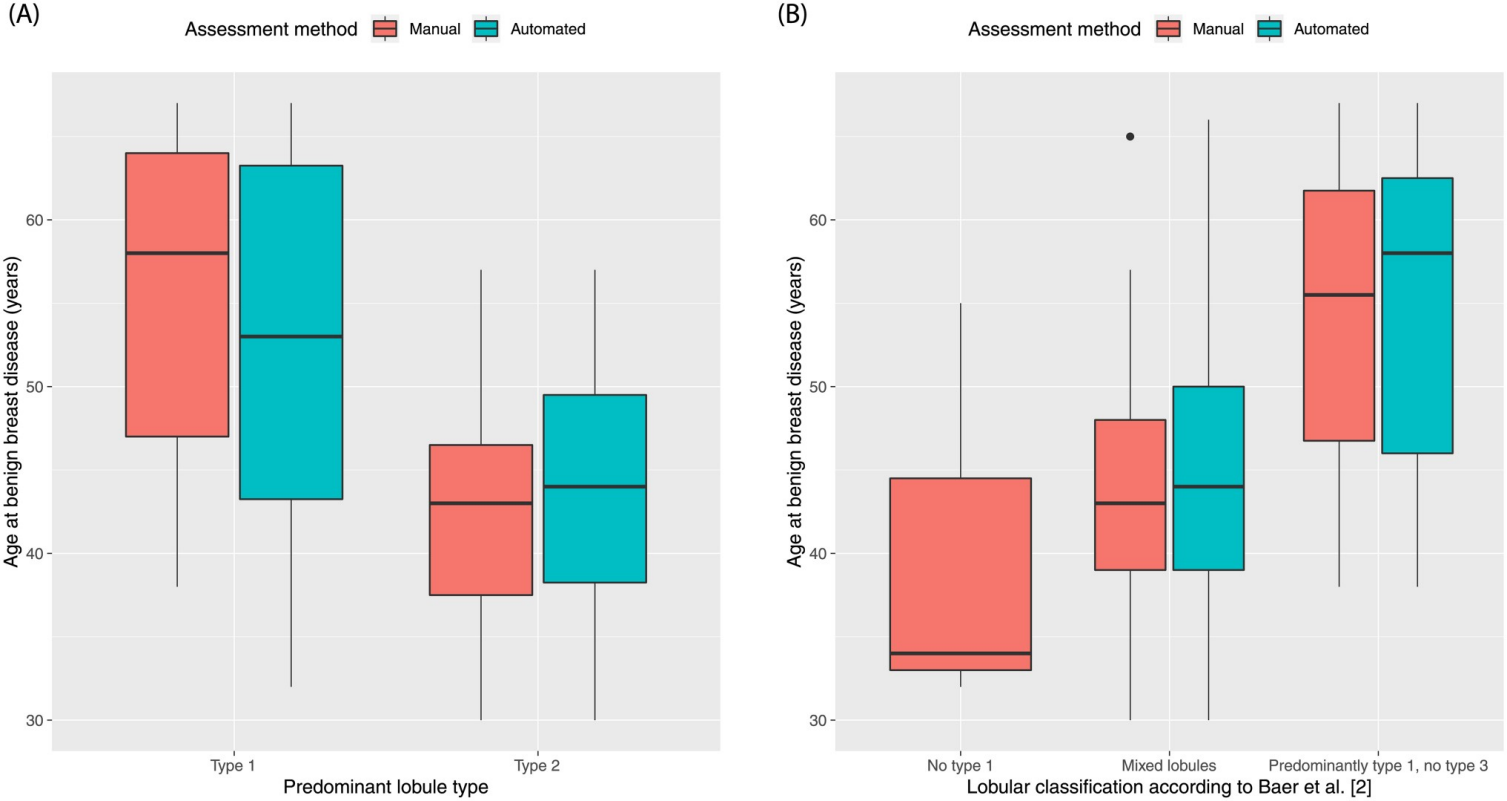
Boxplots demonstrating the association of qualitative terminal ductal lobular unit involution measures and age. (**A)** Women with predominantly type 1 lobules were significantly older than women with predominantly type 2 lobules (manual method: *p*<0.01; automated method: *p* = 0.01). No woman presented with predominately type 3 lobules. (**B**) Women with “Predominantly type 1, no type 3” lobules were significantly older than women with “Mixed lobules” (manual method *p*<0.01; automated method *p*<0.01). No woman was assessed as having “No type 1” lobules by the automated method. The manual qualitative measures were obtained by consensus vote. The boxplots show the median value, interquartile range (IQR), and 5th and 95th whiskers.

All quantitative and qualitative measures obtained by manual and automated methods were significantly associated with age (*p*<0.05; Figs 4 and 5). Table 4 shows the relationships between TDLU measures and menopausal status. All quantitative measures were significantly different between pre- and post-menopausal women, except number of TDLUs per tissue area evaluated by the automated method (*p* = 0.06). Likewise, qualitative measures (consensus vote by observers and automated method) were significantly different between pre- and post-menopausal women, except lobular classification according to Baer *et al*. [2] assessed by the automated method (*p* = 0.07). No participant was classified as predominantly type 3 according to Russo *et al*. [2]. Qualitative measures when assessed by individual observers were not associated with menopausal status (*p*>0.05; S1 Table).

**Table 4:** The association of terminal ductal lobular unit (TDLU) involution measures and menopausal status.

	Pre-Menopausal	Post-Menopausal	p-value
*N*	20	20	
**Quantitative measures**			
Number of TDLU per tissue area (mm^2^), median *n (IQR)*			
Evaluated by observers	0.74 (0.46,1.34)	0.65 (0.27,0.86)	**0.04**
Evaluated by the automated method	1.19 (1.05,1.84)	1.07 (0.92,1.26)	0.06
Median TDLU span in *μ*m, median *n (IQR)*			
Evaluated by observers	740.40 (502.35,810.02)	362.90 (317.01,519.75)	**<0.01**
Evaluated by the automated method	536.64 (504.17,580.56)	448.35 (392.73,587.87)	**<0.05**
Number of acini per TDLU, median *n (IQR)*	29.00 (16.81,48.00)	11.75 (8.50,20.06)	**<0.01**
Evaluated by observers			
Evaluated by the automated method	30.13 (26.24,40.34)	19.44 (13.12,24.30)	**<0.01**
Number of acini per tissue area (mm^2^), median *n (IQR)*	14.18 (6.30,20.09)	5.75 (3.43,8.90)	**<0.01**
Evaluated by the automated method			
Median TDLU area (mm^2^), median *n (IQR)*			
Evaluated by the automated method	0.10 (0.08,0.12)	0.06 (0.06,0.10)	**<0.01**
**Qualitative assessment**			
Predominant lobular type by observers (consensus vote), *n (%)*			**0.01**
Type 1	4 (20.0)	13 (65.0)	
Type 2	16 (80.0)	7 (35.0)	
Type 3	0 (0.0)	0 (0.0)	
Predominant lobular type by the automated method, *n (%)*			**0.02**
Type 1	4 (20.0)	12 (60.0)	
Type 2	16 (80.0)	8 (40.0)	
Type 3	0 (0.0)	0 (0.0)	
Lobular classification according to Baer *et al*. [2] by observers (consensus vote), *n (%)*			**0.04**
No type 1	2 (10.0)	1 (5.0)	
Mixed lobules	14 (70.0)	7 (35.0)	
Predominantly type 1, no type 3	4 (20.0)	12 (60.0)	
Lobular classification according to Baer *et al*. [2] by the automated method, *n (%)*			0.07
No type 1	0 (0.0)	0 (0.0)	
Mixed lobules	18 (90.0)	12 (60.0)	
Predominantly type 1, no type 3	2 (10.0)	8 (4 0.0)	

Thus, older and post-menopausal women had significantly fewer TDLUs/mm^2^, smaller TDLUs, reduced number of acini per TDLU, and fewer acini/mm^2^ compared to pre-menopausal women. Type 1 lobules were predominantly observed in post-menopausal women while the majority of pre-menopausal women had mixed lobules.

## Discussion

Greater amounts of TDLU involution are inversely associated with breast cancer risk [5, 6, 9–11] and aggressive breast cancer subtypes [13, 14]. It is important to better understand TDLU involution as well as epidemiological factors that influence the involution process to obtain deeper insights into breast carcinogenesis and identify new opportunities for breast cancer prevention. A major bottleneck to studying TDLU involution and breast cancer risk in large epidemiological cohorts is the need for manual qualitative and/or quantitative assessment by pathologists. In this study, we developed and validated a computational pathology method that can assess five quantitative and two qualitative measures of TDLU involution. Our automated method was highly comparable to manual assessment, and we confirmed that our TDLU involution measures reflect age and menopausal status [4]. This technology will be a valuable research tool to facilitate future breast cancer risk studies.

Our automated method integrates three separate networks for acini detection, TDLU segmentation, and adipose tissue segmentation. It was challenging to develop the TDLU segmentation network compared to the other two networks because TDLUs have highly variable appearances and BBD encompasses a wide range of morphology. As such, the TDLU segmentation network required more training WSIs to achieve a Dice score similar to the adipose tissue segmentation network. Since we are the first to develop networks for acini detection and TDLU segmentation, we were unable to benchmark our networks. We identified three primary causes of discordance between manual assessment and the automated method which affected our F1 and Dice scores: 1) acini with proliferative or metaplastic changes were frequently detected by the network but were intentionally excluded from manual annotation; 2) the network had difficulty predicting boundaries of TDLUs with complex clustering; and 3) in some cases, the network interpreted large ducts as adipose tissue.

Despite researchers’ best efforts to create a perfect method, most automated methods remain prone to segmentation errors. Solutions to address these issues and improve our computational method include increasing the number of training samples with improved annotation and applying hard negative mining. The inclusion of abnormal epithelium when assessing TDLU involution may influence breast cancer risk assessment. Therefore, future work will evaluate the inter-variability of TDLU measures between slides obtained from different tissue blocks for each patient. In addition, summarizing the automated results using median instead of mean, and evaluating at least two WSIs per case (averaging the median values), will improve the robustness and reliability of the data in future studies. This study focuses on assessing TDLU involution in non-malignant breast tissue only. If this method were to be used to assess TDLU involution in tumor-adjacent normal breast tissues, it would need to be re-trained to include malignant tissue.

To capture TDLU span, the automated method uses the length of the major axis of the ellipse that is identical to the normalized second central moments for each TDLU. In contrast, a pathologist has to select two opposite points along the boundary of a TDLU to obtain the longest span. Thus, the manual assessment of TDLU span inevitably contains some subjectivity and explains the low inter-observer agreement score between manual and automated results. Our automated method has the ability to capture two new measures: number of acini per tissue area and median TDLU area. Future studies will evaluate and compare these newer measures with the existing three standardized measures to determine which TDLU involution quantitative measure is most associated with breast cancer risk.

TDLU involution is historically assessed using qualitative measures [2, 5, 9]. The large variation in intra- and inter-observer Kappa scores as observed in this study reiterated the high subjectivity of qualitative measures, thus spurring researchers to develop standardized quantitative measures to assess TDLU involution [4, 10, 13, 14]. Our study showed higher intra- and inter-observer agreement for quantitative measures compared to qualitative measures, again highlighting the reproducibility of quantitative measures. Despite assessing different tissue areas for manual assessment (observers selected 50 mm^2^ tissue area) and automated method (entire tissue area on WSI), the good agreement between the observers and automated results provided additional assurance that our automated method is comparable to manual assessment.

## Conclusion

We developed and validated an automated method to measure TDLU involution as a first step towards automated prediction of breast cancer risk. Qualitative assessment of TDLU involution is a subjective process. Quantitative assessment produces more reproducible results but is labor-intensive for pathologists. Our method can eliminate the labor-intensiveness and subjectivity of manual TDLU involution assessment. Our technology can be applied on a larger scale to assess breast cancer risk in epidemiological studies. Future work will determine the best quantitative TDLU involution measure to predict breast cancer risk, and evaluate the impact of incorporating these measures into clinical breast cancer risk assessment models to improve patient management.

## Supporting information

S1 Fig Line charts demonstrating the F1 score obtained on the test set with models trained using different percentages of the training dataset. (**A**) The ablation experiment for the detection of acini. The line converges before it reaches 100% of the training data indicating that the training set is large enough. (**B**) The ablation experiment for the segmentation of TDLUs. The line converges before it reaches 100% of the training data indicating that the training set is large enough. The line charts show the mean value and standard deviation.
(DOCX)

S2 Fig Results of the automated method (A.2, B.2, C.2) overlaid on original whole slide images (A.1, B.1, C.1). Detected acini are shown in blue, terminal duct lobular units (TDLUs) in pink, and adipose tissue in yellow. The black crosses (**C.2**) indicate regions where intraductal papillomas were incorrectly segmented as TDLUs.
(DOCX)

S1 Table The association of terminal ductal lobular unit (TDLU) involution measures and menopausal status. (DOCX)

S1 Methods (DOCX)

## References

[1] Wellings SR, Jensen HM, Marcum RG. An atlas of subgross pathology of the human breast with special reference to possible precancerous lesions. J Natl Cancer Inst. 1975;55(2):231–73. 169369

[2] Russo J, Romero AL, Russo IH. Architectural pattern of the normal and cancerous breast under the influence of parity. Cancer Epidemiol Biomarkers Prev. 1994;3:219–24. 8019370

[3] Russo J, Hu YF, Yang X, Russo IH. Chapter 1: Developmental, cellular, and molecular basis of human breast cancer. J Natl Cancer Inst Monographs. 2000;27:17–37. 10.1093/oxfordjournals.jncimonographs.a024241 10963618

[4] Figueroa JD, Pfeiffer RM, Patel DA, Linville L, Brinton LA, Gierach GL, et al Terminal duct lobular unit involution of the normal breast: implications for breast cancer etiology. J Natl Cancer Inst. 2014;106:10. 10.1093/jnci/dju286 PMC4200067 25274491

[5] Milanese TR, Hartmann LC, Sellers TA, Frost MH, Vierkant RA, Maloney SD, et al Age-related lobular involution and risk of breast cancer. J Natl Cancer Inst. 2006;98(22):1600–7. 10.1093/jnci/djj439 17105983

[6] Ginsburg OM, Martin LJ, Boyd NF. Mammographic density, lobular involution, and risk of breast cancer. Br J Cancer. 2008;99:1369–74. 10.1038/sj.bjc.6604635 18781174 PMC2579686

[7] Henson DE, Tarone RE. Involution and the etiology of breast cancer. Cancer. 1994;74:424–29. 10.1002/cncr.2820741330 8004616

[8] Jensen HM. On the origin and progression of human breast cancer. Am J Obstet Gynecol. 1986;154(6):1280–4. 10.1016/0002-9378(86)90713-1 2424309

[9] Baer HJ, Collins LC, Connolly JL, Colditz GA, Schnitt SJ, Tamimi RM. Lobule type and subsequent breast cancer risk: results from the nurses’ health studies. Cancer. 2009;115:1404–11. 10.1002/cncr.24167 19170235 PMC2661011

[10] Figueroa JD, Pfeiffer RM, Brinton LA, Palakal MM, Degnim AC, Radisky D, et al Standardized measures of lobular involution and subsequent breast cancer risk among women with benign breast disease: a nested case–control study. Breast Cancer Res Treat. 2016;159(1):163–72. 10.1007/s10549-016-3908-7 27488681 PMC5045857

[11] McKian KP, Reynolds CA, Visscher DW, Nassar A, Radisky DC, Vierkant RA, et al Novel breast tissue feature strongly associated with risk of breast cancer. J Clin Oncol. 2009;27(35):5893–8. 10.1200/JCO.2008.21.5079 19805686 PMC2793038

[12] Rosebrock A, Caban JJ, Figueroa J, Gierach G, Linville L, Hewitt S, et al. Quantitative analysis of TDLUs using adaptive morphological shape techniques. In: Medical Imaging 2013: Digital Pathology. 2013;8676. International Society for Optics and Photonics. 10.1117/12.2006619 PMC4339025 25722829

[13] Guo C, Sung H, Zheng S, Guida J, Li E, Li J, et al Age-related terminal duct lobular unit involution in benign tissues from Chinese breast cancer patients with luminal and triple-negative tumors. Breast Cancer Res. 2017;19(1):61 10.1186/s13058-017-0850-5 28545469 PMC5445352

[14] Yang XR, Figueroa JD, Falk RT, Zhang H, Pfeiffer RM, Hewitt SM, et al Analysis of terminal duct lobular unit involution in luminal A and basal breast cancers. Breast Cancer Res. 2012;14,:R64 10.1186/bcr3170 22513288 PMC3446399

[15] Dimitriou N, Arandjelović O, Caie PD. Deep Learning for Whole Slide Image Analysis: An Overview. Front Med. 2019;6. 10.3389/fmed.2019.00264 PMC6882930 31824952

[16] Harder N, Schönmeyer R, Nekolla K, Meier A, Brieu N, Vanegas C, et al Automatic discovery of image-based signatures for ipilimumab response prediction in malignant melanoma. Sci Rep. 2019;9(1):7449 10.1038/s41598-019-43525-8 31092853 PMC6520405

[17] Brieu N, Gavriel CG, Nearchou IP, Harrison DJ, Schmidt G, Caie PD. Automated tumour budding quantification by machine learning augments TNM staging in muscle-invasive bladder cancer prognosis. Sci Rep. 2019;9(1):5174 10.1038/s41598-019-41595-2 30914794 PMC6435679

[18] Caie PD, Zhou Y, Turnbull AK, Oniscu A, Harrison DJ. Novel histopathologic feature identified through image analysis augments stage II colorectal cancer clinical reporting. Oncotarget. 2016;7(28):44381 10.18632/oncotarget.10053 27322148 PMC5190104

[19] Beck AH, Sangoi AR, Leung S, Marinelli RJ, Nielsen TO, Van De Vijver MJ, et al Systematic analysis of breast cancer morphology uncovers stromal features associated with survival. Sci Transl Med. 2011;3(108):108ra113-. 10.1126/scitranslmed.3002564 22072638

[20] Nearchou IP, Lillard K, Gavriel CG, Ueno H, Harrison DJ, Caie PD. Automated Analysis of Lymphocytic Infiltration, Tumor Budding, and Their Spatial Relationship Improves Prognostic Accuracy in Colorectal Cancer. Cancer Immunol Res. 2019;7(4):609–20. 10.1158/2326-6066.CIR-18-0377 30846441

[21] Xu Y, Mo T, Feng Q, Zhong P, Lai M, Eric I, et al. Deep learning of feature representation with multiple instance learning for medical image analysis. In 2014 IEEE international conference on acoustics, speech and signal processing (ICASSP). 2014;1626–1630. IEEE.

[22] Bulten W, Pinckaers H, van Boven H, Vink R, de Bel T, van Ginneken B, et al Automated deep-learning system for Gleason grading of prostate cancer using biopsies: a diagnostic study. The Lancet Oncology. 2020. 10.1016/S1470-2045(19)30739-9 31926805

[23] Chang H, Han J, Zhong C, Snijders AM, Mao JH. Unsupervised transfer learning via multi-scale convolutional sparse coding for biomedical applications. In 2017 IEEE transactions on pattern analysis and machine intelligence. 2017;40(5):1182–94. IEEE. 10.1109/TPAMI.2017.2656884 28129148 PMC5522776

[24] Ertosun MG, Rubin DL. Automated grading of gliomas using deep learning in digital pathology images: A modular approach with ensemble of convolutional neural networks. In AMIA Annual Symposium Proceedings 2015. 2015;1899–908. American Medical Informatics Association. PMC4765616 26958289

[25] Källén H, Molin J, Heyden A, Lundström C, Åström K. Towards grading gleason score using generically trained deep convolutional neural networks. In 2016 IEEE 13th International Symposium on Biomedical Imaging (ISBI). 2016;1163–67. IEEE.

[26] Litjens G, Sánchez CI, Timofeeva N, Hermsen M, Nagtegaal I, Kovacs I, et al Deep learning as a tool for increased accuracy and efficiency of histopathological diagnosis. Sci Rep. 2016;6:26286 10.1038/srep26286 27212078 PMC4876324

[27] Yue X, Dimitriou N, Caie DP, Harrison JD, Arandjelovic O. Colorectal cancer outcome prediction from H&E whole slides images using machine learning and automatically inferred phenotype profiles. In Conference on Bioinformatics and Computational Biology. 2019;60:139–49.

[28] Veta M, Heng YJ, Stathonikos N, Bejnordi BE, Beca F, Wollmann T, et al Predicting breast tumor proliferation from whole-slide images: the TUPAC16 challenge. Med Image Anal. 2019;54:111–21. 10.1016/j.media.2019.02.012 30861443

[29] Wetstein SC, Onken AM, Baker GM, Pyle ME, Pluim JP, Tamimi RM, et al. Detection of acini in histopathology slides: towards automated prediction of breast cancer risk. In: Medical Imaging 2019: Digital Pathology. 2019;10956. International Society for Optics and Photonics.

[30] Bejnordi BE, Mullooly M, Pfeiffer RM, Fan S, Vacek PM, Weaver DL, et al Using deep convolutional neural networks to identify and classify tumor-associated stroma in diagnostic breast biopsies. Mod Pathol. 2018;31(10):1502 10.1038/s41379-018-0073-z 29899550 PMC6752036

[31] Balkenhol MCA, Tellez D, Vreuls W, Clahsen PC, Pinckaers H, Ciompi F, et al Deep learning assisted mitotic counting for breast cancer. Lab Invest. 2019;99(11):1596–606. 10.1038/s41374-019-0275-0 31222166

[32] Veta M, Van Diest PJ, Jiwa M, Al-Janabi S, Pluim JPW. Mitosis counting in breast cancer: Object-level interobserver agreement and comparison to an automatic method. PloS one. 2016;11(8):e0161286 10.1371/journal.pone.0161286 27529701 PMC4987048

[33] Wang D, Khosla A, Gargeya R, Irshad H, Beck AH. Deep learning for identifying metastatic breast cancer. arXiv: 1606.05718. 2016.

[34] Bejnordi BE, Veta M, Van Diest PJ, Van Ginneken B, Karssemeijer N, Litjes G, et al Diagnostic assessment of deep learning algorithms for detection of lymph node metastases in women with breast cancer. JAMA. 2017;318(22):2199–210. 10.1001/jama.2017.14585 29234806 PMC5820737

[35] Colditz GA, Hankinson SE. The Nurses' Health Study: lifestyle and health among women. Nat Rev Cancer. 2005;5(5):388–96. 10.1038/nrc1608 15864280

[36] Tamimi RM, Byrne C, Baer HJ, Rosner B, Schnitt SJ, Connolly JL, et al Benign breast disease, recent alcohol consumption, and risk of breast cancer: a nested case–control study. Breast Cancer Res. 2005;7(4):R555–62. 10.1186/bcr1039 15987462 PMC1175067

[37] Collins LC, Baer HJ, Tamimi RM, Connolly JL, Colditz GA, Schnitt SJ. The influence of family history on breast cancer risk in women with biopsy-confirmed benign breast disease: results from the Nurses' Health Study. Cancer. 2006;107(6):1240–7. 10.1002/cncr.22136 16902983

[38] Collins LC, Baer HJ, Tamimi RM, Connolly JL, Colditz GA, Schnitt SJ. Magnitude and laterality of breast cancer risk according to histologic type of atypical hyperplasia: results from the Nurses' Health Study. Cancer. 2007;109(2):180–7. 10.1002/cncr.22408 17154175

[39] Tamimi RM, Colditz GA, Wang Y, Collins LC, Hu R, Rosner B, et al Expression of IGF1R in normal breast tissue and subsequent risk of breast cancer. Breast Cancer Res Treat. 2011;128(1):243–50. 10.1007/s10549-010-1313-1 21197570 PMC3116083

[40] Aroner SA, Collins LC, Connolly JL, Colditz GA, Schnitt SJ, Rosner BA, et al Radial scars and subsequent breast cancer risk: results from the Nurses’ Health Studies. Breast Cancer Res Treat. 2013;139(1):277–85. 10.1007/s10549-013-2535-9 23609472 PMC3689547

[41] Collins LC, Aroner SA, Connolly JL, Colditz GA, Schnitt SJ, Tamimi RM. Breast cancer risk by extent and type of atypical hyperplasia: An update from the Nurses' Health Studies. Cancer. 2016;122(4):515–20. 10.1002/cncr.29775 26565738 PMC4742394

[42] Kensler KH, Beca F, Baker GM, Heng YJ, Back AH, Schnitt SJ, et al Androgen receptor expression in normal breast tissue and subsequent breast cancer risk. NPJ Breast Cancer. 2018;4(1):33. 30276234 10.1038/s41523-018-0085-3 PMC6155011

[43] Long J, Shelhamer E, Darrell T. Fully convolutional networks for semantic segmentation. In: In Proceedings of the IEEE Conference on Computer Vision and Pattern Recognition. 2015:3431–40. 10.1109/TPAMI.2016.2572683 27244717

[44] Ronneberger O, Fischer P, Brox, T. U-net: Convolutional networks for biomedical image segmentation. In: Springer, C. (ed.) International Conference on Medical Image Computing and Computer-assisted Intervention. 2015:234–41.

[45] Koo TK, Li MY. A guideline of selecting and reporting intraclass correlation coefficients for reliability research. J Chiropr Med. 2016;15:155–63. 10.1016/j.jcm.2016.02.012 27330520 PMC4913118

