# Supplementary material for: Deep learning assessment of breast terminal duct lobular unit involution: Towards automated prediction of breast cancer risk
Source: PLoS One. 2020 Apr 15;15(4):e0231653. doi: 10.1371/journal.pone.0231653 (PMC7159218; doi:10.1371/journal.pone.0231653)
Supplement: S1 Fig — (A) The ablation experiment for the detection of acini. The line converges before it reaches 100% of the training data indicating that the training set is large enough. (B) The ablation experiment for the segmentation of TDLUs. The line converges before it reaches 100% of the training data indicating that the training set is large enough. The line charts show the mean value and standard deviation. (DOCX) [file pone.0231653.s001.docx]

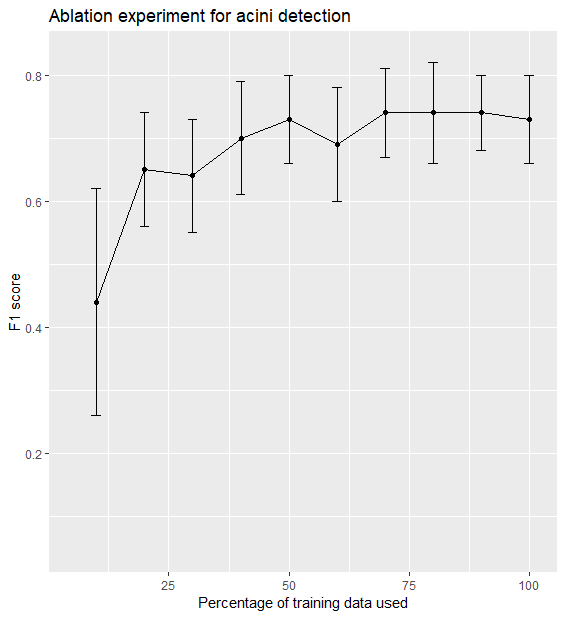

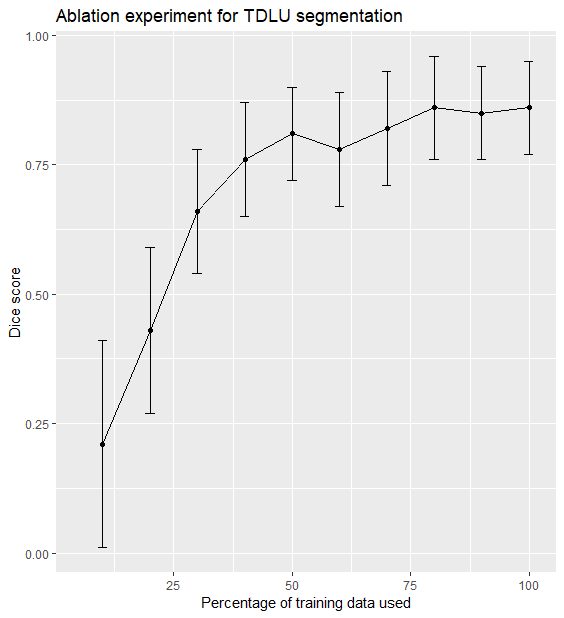


**A**

**B**

**S1 Figure:** Line charts demonstrating the F1 score obtained on the test set with models trained using different percentages of the training dataset. (**A**) The ablation experiment for the detection of acini. The line converges before it reaches 100% of the training data indicating that the training set is large enough. (**B**) The ablation experiment for the segmentation of TDLUs. The line converges before it reaches 100% of the training data indicating that the training set is large enough. The line charts show the mean value and standard deviation.
