# Supplementary material for: Deep learning assessment of breast terminal duct lobular unit involution: Towards automated prediction of breast cancer risk
Source: PLoS One. 2020 Apr 15;15(4):e0231653. doi: 10.1371/journal.pone.0231653 (PMC7159218; doi:10.1371/journal.pone.0231653)
Supplement: S2 Fig — Detected acini are shown in blue, terminal duct lobular units (TDLUs) in pink, and adipose tissue in yellow. The black crosses (C.2) indicate regions where intraductal papillomas were incorrectly segmented as TDLUs. (DOCX) [file pone.0231653.s002.docx]

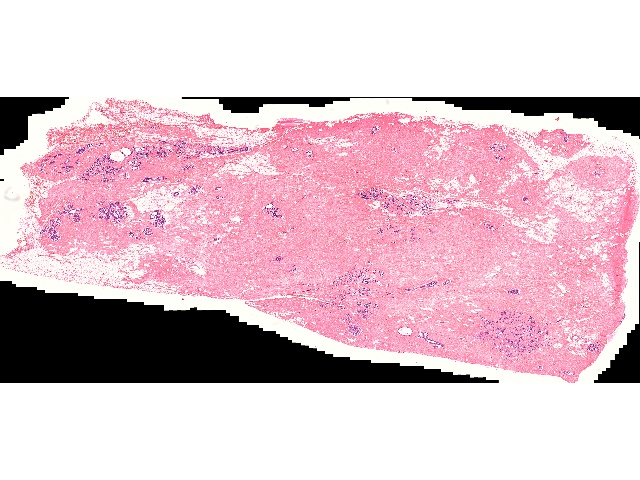


**A.1**

**A**


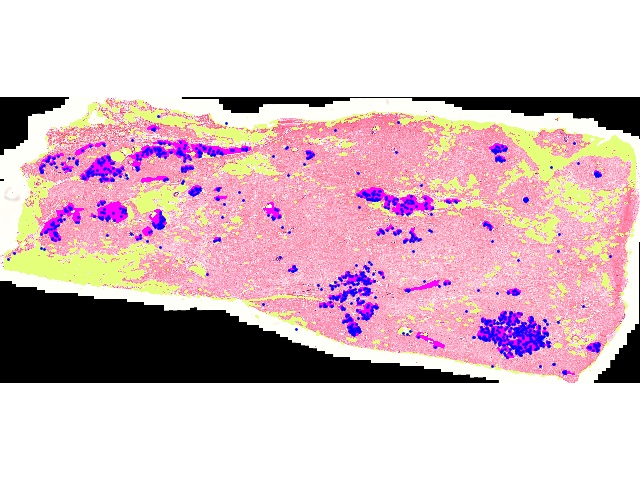


Acini

TDLU

Adipose

tissue

**A.2**

**A.1**


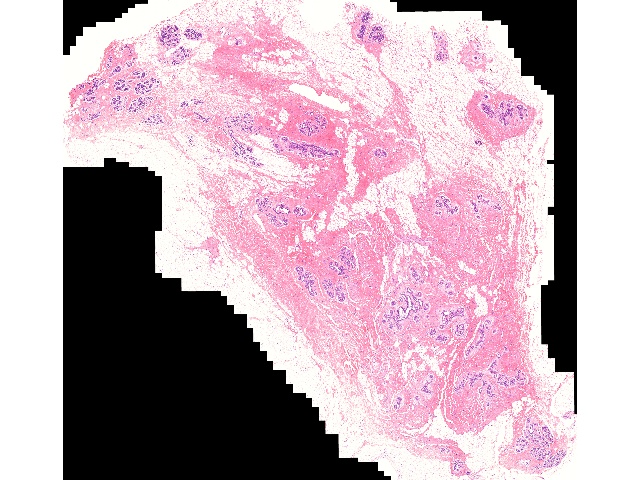


**B.1**


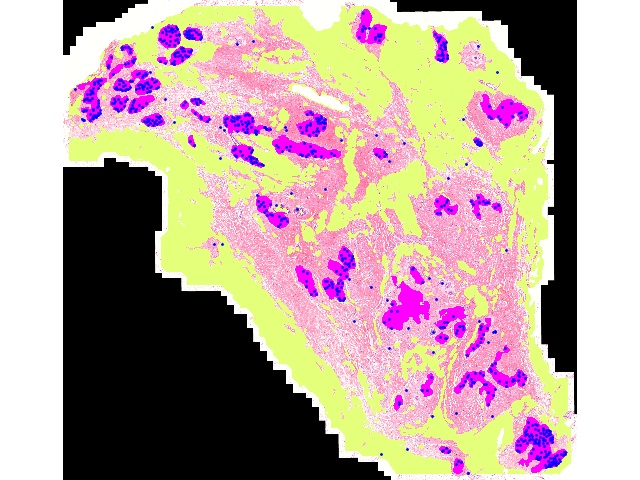


Acini

TDLU

Adipose

tissue

**B.2**


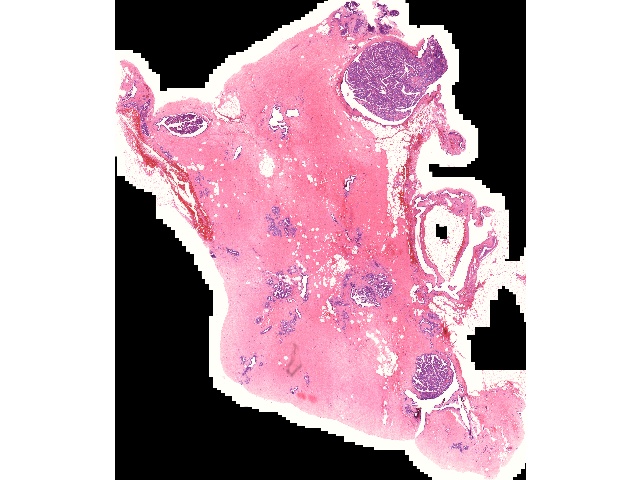


**C.1**


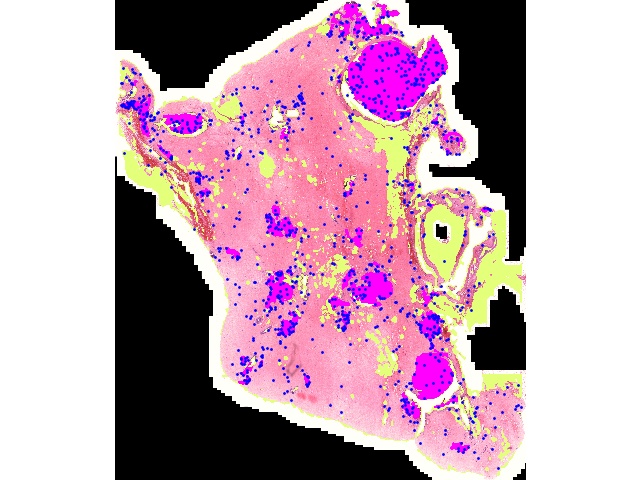


**C.2**

Incorrect TDLU segmentation

Acini

TDLU

Adipose tissue

**S2 Figure:** Results of the automated method (**A.2, B.2, C.2**) overlaid on original whole slide images (**A.1, B.1, C.1**). Detected acini are shown in blue, terminal duct lobular units (TDLUs) in pink, and adipose tissue in yellow. The black crosses (**C.2**) indicate regions where intraductal papillomas were incorrectly segmented as TDLUs.
