# Supplementary material for: Deep learning assessment of breast terminal duct lobular unit involution: Towards automated prediction of breast cancer risk
Source: PLoS One. 2020 Apr 15;15(4):e0231653. doi: 10.1371/journal.pone.0231653 (PMC7159218; doi:10.1371/journal.pone.0231653)
Supplement: S1 Table — (DOCX) [file pone.0231653.s003.docx]

**S1 Table:** The association of terminal ductal lobular unit (TDLU) involution measures and menopausal status.

|  | Pre-Menopausal | Post-Menopausal | p-value |
| --- | --- | --- | --- |
| *n* | 20 | 20 |  |
| **Quantitative measures**  Number of TDLU per tissue area (mm^2^), median *n (IQR)*  Evaluated by observers | 0.74 (0.46,1.34) | 0.65 (0.27,0.86) | **0.04** |
| Evaluated by the automated method | 1.19 (1.05,1.84) | 1.07 (0.92,1.26) | 0.06 |
| Median TDLU span in *µ*m, median *n (IQR)*  Evaluated by observers | 740.40 (502.35,810.02) | 362.90 (317.01,519.75) | ***<*0.01** |
| Evaluated by the automated method | 536.64 (504.17,580.56) | 448.35 (392.73,587.87) | **<0.05** |
| Number of acini per TDLU, median *n (IQR)* Evaluated by observers | 29.00 (16.81,48.00) | 11.75 (8.50,20.06) | ***<*0.01** |
| Evaluated by the automated method | 30.13 (26.24,40.34) | 19.44 (13.12,24.30) | ***<*0.01** |
| Number of acini per tissue area (mm^2^), median *n (IQR)*  Evaluated by the automated method | 14.18 (6.30,20.09) | 5.75 (3.43,8.90) | ***<*0.01** |
| Median TDLU area (mm^2^), median *n (IQR)*  Evaluated by the automated method | 0.10 (0.08,0.12) | 0.06 (0.06,0.10) | ***<*0.01** |
| **Qualitative assessment**  Predominant lobular type by observer 1, *n (%)* |  |  | 0.13 |
| Type 1 | 6 (30.0) | 12 (60.0) |  |
| Type 2 | 13 (65.0) | 6 (30.0) |  |
| Type 3 | 1 (5.0) | 1 (5.0) |  |
| Missing | 0 (0.0) | 1 (5.0) |  |
| Predominant lobular type by observer 2, *n (%)* |  |  | 0.05 |
| Type 1 | 4 (20.0) | 11 (55.0) |  |
| Type 2 | 16 (80.0) | 9 (45.0) |  |
| Type 3 | 0 (0.0) | 0 (0.0) |  |
| Predominant lobular type by observer 3, *n (%)* |  |  | 0.07 |
| Type 1 | 6 (30.0) | 12 (60.0) |  |
| Type 2 | 14 (70.0) | 7 (35.0) |  |
| Type 3 | 0 (0.0) | 0 (0.0) |  |
| Missing | 0 (0.0) | 1 (5.0) |  |
| Predominant lobular type by observers (consensus vote), *n (%)* |  |  | **0.01** |
| Type 1 | 4 (20.0) | 13 (65.0) |  |
| Type 2 | 16 (80.0) | 7 (35.0) |  |
| Type 3 | 0 (0.0) | 0 (0.0) |  |
| Predominant lobular type by the automated method, *n (%)* |  |  | **0.02** |
| Type 1 | 4 (20.0) | 12 (60.0) |  |
| Type 2 | 16 (80.0) | 8 (40.0) |  |
| Type 3 | 0 (0.0) | 0 (0.0) |  |
| Lobular classification according to Baer *et al.* [2] by observer 1, *n (%)* |  |  | 0.24 |
| No Type 1 | 2 (10.0) | 1 (5.0) |  |
| Mixed lobules | 13 (65.0) | 8 (40.0) |  |
| Predominantly Type 1, no Type 3 | 5 (25.0) | 9 (45.0) |  |
| Missing data | 0 (0.0) | 1 (5.0) |  |
| Lobular classification according to Baer *et al.* [2] by observer 2, *n (%)* |  |  | ***<*0.01** |
| No Type 1 | 11 (55.0) | 1 (5.0) |  |
| Mixed lobules | 5 (25.0) | 9 (45.0) |  |
| Predominantly Type 1, no Type 3 | 4 (20.0) | 10 (50.0) |  |
| Lobular classification according to Baer *et al.* [2] by observer 3, *n (%)* |  |  | **0.03** |
| No Type 1 | 0 (0.0) | 0 (0.0) |  |
| Mixed lobules | 15 (75.0) | 7 (35.0) |  |
| Predominantly Type 1, no Type 3 | 5 (25.0) | 12 (60.0) |  |
| Missing data | 0 (0.0) | 1 (5.0) |  |
| Lobular classification according to Baer *et al.* [2] by observers (consensus vote), *n (%)* |  |  | **0.04** |
| No Type 1 | 2 (10.0) | 1 (5.0) |  |
| Mixed lobules | 14 (70.0) | 7 (35.0) |  |
| Predominantly Type 1, no Type 3 | 4 (20.0) | 12 (60.0) |  |
| Lobular classification according to Baer *et al.* [2] by the automated method, *n (%)* |  |  | 0.07 |
| No Type 1 | 0 (0.0) | 0 (0.0) |  |
| Mixed lobules | 18 (90.0) | 12 (60.0) |  |
| Predominantly Type 1, no Type 3 | 2 (10.0) | 8 (40.0) |  |
