# Supplementary material for: Deep learning assessment of breast terminal duct lobular unit involution: Towards automated prediction of breast cancer risk
Source: PLoS One. 2020 Apr 15;15(4):e0231653. doi: 10.1371/journal.pone.0231653 (PMC7159218; doi:10.1371/journal.pone.0231653)
Supplement: S1 Methods — (DOCX) [file pone.0231653.s004.docx]

**S1 Methods**

**Convolutional neural network architecture**

The neural network used to train our acini detection, TDLU segmentation and adipose tissue segmentation networks is similar to the one described by Ronneberger *et al*. [1]. We use U-Nets with the same depth and amount of filters. The U-Net architecture consists of a contracting path (as is usual in neural networks) succeeded by a symmetric expanding path. This architecture was designed to capture context yet also enable precise localization of objects. As described by Falk et al. [2], U-net applies to general pixel-classification tasks and can therefore be used for segmentation, as well as detection tasks.

During training patches of 512×512 pixels were extracted from the annotated part of the WSI and randomly translated and rotated. The mini batch size was set to 10 patches and the network was trained by minimizing the binary cross-entropy between the ground truth and predictions with an Adam optimizer with a learning rate of 1e-6. Training was stopped when the average of the validation loss over 10 epochs increased. Hyper parameters like the depth of the U-Net, the amount of filters, the mini batch size and the learning rate were tuned to optimal performance on the validation set by grid search. This was done for each network individually but the same parameters were optimal for all three.

**Acini detection**

The acini detection network was trained and evaluated using 50 WSIs from 50 NHS/NHSII participants. A five-way split into training, validation and test sets was used. In each of the five splits, 30 WSIs were used for training, 10 for validation and 10 for testing. In these WSIs a region comprising 10% of the total tissue area was annotated. The annotations were centroid only, meaning that the center pixel was annotated and not the extent of the acinus. Since only the center pixel of the acini was annotated, taking the trivial segmentation approach would lead to a severe class imbalance. To address this, we defined alternative targets to train our deep learning architecture on. A comparison of different targets can be found in our previous work [3]. The best performing method was to use soft centroid labels, in which we place an isotropic Gaussian with a standard deviation of 10 pixels at the location of each acinus centroid.

After training, the predicted target maps were converted to acini centroid predictions by using non-maximum suppression in a radius of 20 pixels, with a threshold of 0.48 (out of 1). These hyper-parameters were determined based on the validation set.

Essentially, the Gaussian label defined a centroid region for each acinus and we segmented this region with a U-Net model. From the resulting segmentations we inferred the location of the acini centroids using non-maximum suppression. After applying non-maximum suppression we were left with a map of points where the acini centroids are predicted to be. The resulting acini maps were then compared with the

ground truth points. In case there was at least one predicted acinus within 20 pixels of a ground truth acinus this was counted as a true positive. In case there was more than one predicted acinus within 20 pixels it was still only counted as one true positive. The total number of true positives was found by comparing every ground truth acinus with our prediction results. We calculated the number of false positives as the total number of predicted acini minus the true positives. False negatives were calculated by subtracting the total number of true positives from the total number of ground truth acini. We calculated the F1 score using these true positives, false positives and false negatives. More information on the acini detection method can be found in [3].

**TDLU segmentation**

The TDLU segmentation network was trained and evaluated using 92 WSIs from 92 NHS/NHSII participants. A nine-way split of the WSIs into sets of 10 (or 11) WSIs was applied. For each fold, 7 sets were used for training (~72 WSIs), 1 set (~10 WSIs) was used for validation and 1 set (~10 WSIs) for testing. In these WSIs a region comprising 10% of the total tissue area was annotated with TDLU segmentations. TDLUs were defined as clusters of acini in a lobular configuration. TDLU boundary was defined by the non-specialized/extra-lobular stroma. In order to assess involution in histologically normal breast parenchyma only, TDLUs with proliferative or metaplastic changes were not annotated.

After training, the predicted target maps were converted to TDLU segmentations by using thresholding and morphological operations. More specifically, we first calculated a threshold for the prediction map using Otsu’s method [4]. All values below this threshold were set to 0. Then, we removed all connected objects in the image that had an area smaller than 2500 pixels. After that, a median filter with a kernel size of 11 was used to remove noise. Lastly, holes in the remaining objects were removed if they were smaller than 2500 pixels. These thresholding and morphological operations and their parameters were determined based on performance on the validation set.

**Adipose tissue segmentation**

The adipose tissue segmentation network was trained and evaluated using 50 WSIs from 50 NHS/NHSII participants A five-way split into training, validation and test sets was used. In each of the five splits, 30 WSIs were used for training, 10 for validation and 10 for testing. In these WSIs a region comprising 2.5% of the total tissue area was annotated with adipose tissue segmentations.

After training, the predicted target maps were converted to adipose tissue segmentations by thresholding with a value of 0.6. This threshold was determined based on performance on the validation set.

**References**

1. Ronneberger O, Fischer P, Brox, T. U-net: Convolutional networks for biomedical image segmentation. In: Springer, C. (ed.) International Conference on Medical Image Computing and Computer-assisted Intervention. 2015:234–41.
2. Falk T, Mai D, Bensch R, Çiçek Ö, Abdulkadir A, Marrakchi Y, et al. U-Net: deep learning for cell counting, detection, and morphometry. Nature methods. 2019 Jan;16(1):67-70.
3. Wetstein SC, Onken AM, Baker GM, Pyle ME, Pluim JP, Tamimi RM, et al. Detection of acini in histopathology slides: towards automated prediction of breast cancer risk. In: Medical Imaging 2019: Digital Pathology. 2019;10956. International Society for Optics and Photonics.
4. Otsu N. A threshold selection method from gray-level histograms. IEEE transactions on systems, man, and cybernetics. 1979 Jan;9(1):62-6.
